# Analysis of multispectral polarization imaging image information based on micro-polarizer array

**DOI:** 10.1371/journal.pone.0296397

**Published:** 2024-01-30

**Authors:** Qiang Fu, Ninglan Ma, Xuanwei Liu, Yue Zhang, Juntong Zhan, Su Zhang, Jin Duan, Yingchao Li

**Affiliations:** 1 College of Opto-Electronic Engineering, Changchun University of Science and Technology, Changchun, China; 2 Space Opto-Electronics Technology Institute, Changchun University of Science and Technology, Changchun, China; TU Wien: Technische Universitat Wien, AUSTRIA

## Abstract

As a new detection technology, polarization imaging is of great significance in the field of target detection. At present, polarization imaging technology usually adopts visible light polarization imaging. The technique is difficult to image the target in complex background due to its narrow working spectrum and short detection distance. Therefore, based on the principle of full Stokes micro-polarizer array, this paper proposes a multi-spectral polarization imaging scheme and designs a multi-spectral polarization imaging detection system penetrating haze. Conducting indoor and outdoor polarized imaging experiments. Finally, image quality was assessed using metrics such as information entropy (EN), average gradient (AG), and standard deviation (STD). The results show that compared with traditional strength detection, the imaging system has significantly improved detection distance and imaging quality in smoky environments. The imaging system can effectively enhance the contours and details of the target object and improve detection and recognition capabilities.

## Introduction

Polarization imaging detection technology is a novel optoelectronic detection technique that possesses unique advantages in target detection. Research on polarization imaging detection technology began in the 1970s in the United States. Over time, five different polarization imaging devices have been developed based on various technical schemes and core components [1–5]. Currently, the field of polarization imaging detection technology is developing rapidly [6–9]. In terms of spectral polarization detection, researchers have conducted polarization imaging experiments at different wavelength ranges. Additionally, new detection technologies specifically designed for spectral polarization imaging have been developed [10–12].

In 2022, China University of Mining and Technology proposed a new image dehazing algorithm based on polarization information and deep prior learning [13]. This method achieved a 31% improvement in contrast for heavily hazy images, providing a new direction for image dehazing processing. In 2023, North China University of Technology proposed the principle of dual-channel complementary snapshot polarization spectral imaging and established a dual-channel multispectral and hyperspectral image fusion model [14]. This approach enhances the spectral and spatial resolution of snapshot polarization spectral imaging, resulting in a 3 dB improvement in spatial resolution.

In recent years, the development of micro-polarizer arrays has become a hot topic in the field of polarization imaging. By adjusting the state of the micro-polarizer array, the polarization information on the surface of the target object can be extracted. It has been widely applied in material characterization, surface topography measurement, and image enhancement [15–17]. In 2018, Sony introduced a visible light zoomable focal plane polarization detector [18]. By integrating a microlens between each pixel and the micro-polarizer, crosstalk of polarized light in different directions is reduced. In 2022, researchers at Harvard University designed a metasurface diffraction grating based on the principle of selective spectral splitting according to the polarization state [19]. This can convert intensity imaging into full Stokes polarization imaging.

Many research efforts in recent years have focused on improving contrast of target images through algorithms such as deep learning, without enhancing imaging performance through advancements in detection devices. Existing detection technologies often employ linear polarization imaging, neglecting the impact of circular polarization on detection capability, and have limited spectral range and low energy utilization.

In complex environments, reflection and scattering of light severely affect the clarity and contrast of images. Linear polarization imaging only selects the vibration direction of light in one direction. When there is an angular deviation between the light and the target surface, the target’s optical properties and structure may exhibit anisotropy, making it impossible to completely eliminate the effects of reflection and scattered light. On the other hand, circular polarization imaging rotates the vibration direction of light, keeping a fixed phase difference between the sample and the vibration direction of light, reducing sensitivity to reflection and scattered light and effectively reducing interference. Therefore, circular polarization exhibits superior polarization preservation capabilities compared to linear polarization in environments with significant optical thickness, and utilizing circular polarization enhancement can improve the imaging quality of targets in complex environments.

Multispectral polarization imaging combines spectral and polarization information, enabling simultaneous acquisition of spectral and polarizing characteristics of samples. By capturing and analyzing data in different spectral bands and polarization states, rich multidimensional information can be obtained, offering a comprehensive description of sample features and properties.

Research efforts have been conducted on micro-nano wire grids and micro-nano circular grids, designing and fabricating micro-polarizer arrays embedded with circular polarization information. Real-time detection of full Stokes polarization spectral multi-parameters has been achieved in the micro-polarizer array, and imaging errors in the micro-polarizer array have been analyzed and corrected. Furthermore, a multispectral polarization imaging detection system has been designed, and experiments of multispectral polarization light transmission imaging have been conducted on targets in indoor simulated and outdoor real environments. The results have demonstrated that compared to conventional imaging detection methods, the multispectral polarization imaging haze detector based on the micro-polarizer array has achieved a 4-fold improvement in detection distance and a 30% increase in image contrast.

These research efforts provide new insights and approaches for the development of polarization imaging technology, achieving improved imaging results in complex environments. Further research and improvements can be expected to bring more breakthroughs and advancements in the field of polarization imaging.

## Basic principles of polarization imaging

The Stokes vector describes the polarization state of the light wave through ${\left(S_{0},S_{1},S_{2},S_{3}\right)}^{T}$, which can also be written as ${\left(I,Q,U,V\right)}^{T}$, where I represents the total light intensity, and Q represents the intensity difference between horizontally and vertically linearly polarized light. U represents the intensity difference between linearly polarized light with different angles, and V represents the intensity difference between right-handed and left-handed circularly polarized light.

When the polarizer is used to detect the intensity of linearly polarized light at any angle, the following relationship can be obtained [20]:

$$\left[\begin{array}{l} I^{\prime }(\theta )\\ Q^{\prime }(\theta )\\ U^{\prime }(\theta )\\ V^{\prime }(\theta ) \end{array}]=M_{p}*[\begin{array}{l} I\\ Q\\ U\\ V \end{array}\right]$$ (1)

Where *M*_*P*_ is the reflection Mueller matrix, bringing *M*_*p*_ into Eq (1) yields

$$\left[\begin{array}{l} I^{\prime }(\theta )\\ Q^{\prime }(\theta )\\ U^{\prime }(\theta )\\ V^{\prime }(\theta ) \end{array}]=\frac{1}{2}[\begin{array}{cccc} 1 & \cos2\theta & \sin2\theta & 0\\ \cos2\theta & \cos^{2}2\theta & \cos2\theta \sin2\theta & 0\\ \sin2\theta & \cos2\theta \sin2\theta & \sin^{2}2\theta & 0\\ 0 & 0 & 0 & 0 \end{array}][\begin{array}{l} I\\ Q\\ U\\ V \end{array}\right]$$ (2)

Where $I^{\prime }(\theta )=\frac{1}{2}(I+Q\cos2\theta +U\sin2\theta )$ is the angle of the polarizer *θ* of the detected light intensity value. The Stokes matrix is a 4th-order matrix and requires the measurement of 4 parameters to obtain the corresponding Stokes matrix. In most natural cases, circular polarization can be ignored and does not affect the detection effect. Therefore, the Stokes matrix can be dimensionally reduced into a 3rd-order matrix, and only 3 parameters need to be measured to solve the Stokes matrix. Using the 0°, 45°, and 90° angles can provide a comprehensive description of the polarimetric properties of the target in different directions. By combining these angles, detailed information about the target can be obtained, resulting in more accurate and complete polarimetric imaging results. Therefore, the 0°, 45°, and 90° linear polarization directions are selected as the parameters to solve the Stokes matrix. When using a single polarizer to detect a target, only the linear polarization information of the target can be obtained, and circular polarization information cannot be acquired. However, when using a combination of wave plate and polarizer, not only can the linear polarization information of the target be detected, but also the circular polarization information can be obtained. Circular polarization has a pronounced effect on penetrating smoke. Therefore, by combining wave plates and polarizing films, better detection results can be achieved for targets in a smoky environment. Then there is [21]:

$$\begin{array}{c} \;\left[\begin{array}{c} I^{\prime }(\theta^{\prime })\\ Q^{\prime }(\theta^{\prime })\\ U^{\prime }(\theta^{\prime })\\ V^{\prime }(\theta^{\prime }) \end{array}]=M_{p}(0^{\circ })*M_{R}\left(\frac{\pi }{2},\theta^{\prime }\right)*[\begin{array}{c} I\\ Q\\ U\\ V \end{array}]=M*M_{R}\left(\frac{\pi }{2},\theta^{\prime }\right)*[\begin{array}{c} I\\ Q\\ U\\ V \end{array}\right]\\ =\frac{1}{2}[\begin{array}{cccc} 1 & 1 & 0 & 0\\ 1 & 1 & 0 & 0\\ 0 & 0 & 0 & 0\\ 0 & 0 & 0 & 0 \end{array}][\begin{array}{cccc} 1 & 0 & 0 & 0\\ 0 & \cos^{2}2\theta^{\prime } & \sin2\theta^{\prime }\cos2\theta^{\prime } & -\sin2\theta^{\prime }\\ 0 & \sin2\theta^{\prime }\cos2\theta^{\prime } & \sin^{2}2\theta^{\prime } & \cos\theta^{\prime }\\ 0 & \sin2\theta^{\prime } & -\cos\theta^{\prime } & 0 \end{array}][\begin{array}{c} I\\ Q\\ U\\ V \end{array}] \end{array}$$ (3)

Where $I^{\prime }(\theta^{\prime })=\frac{1}{2}(I+Q\cos^{2}2\theta^{\prime }+U\sin2\theta^{\prime }\cos2\theta^{\prime }-V\sin2\theta^{\prime })$ denotes a polarizer angle of 0° and the angle of the waveplate is *θ*′,

we choose the value of the detected light intensity when *θ*′ values as $\frac{\pi }{4}$ with $-\frac{\pi }{4}$, when $\theta^{\prime }=\frac{\pi }{4}$ the detected light intensity value is denoted by $I_{l}^{\prime }$ is expressed; when $\theta^{\prime }=-\frac{\pi }{4}$ the detected light intensity is denoted by $I_{r}^{\prime }$ and the Stokes vector is obtained by calculating $V=I_{r}^{\prime }-I_{l}^{\prime }$. From this, the Stokes expression is obtained as [21]:

$$\left[\begin{array}{l} I\\ Q\\ U\\ V \end{array}]=[\begin{array}{c} 2/3\times (I^{\prime }(0^{\circ })+I^{\prime }(45^{\circ })+I^{\prime }(90^{\circ }))\\ 2/3\times (2\times I^{\prime }(0^{\circ })-I^{\prime }(45^{\circ })-I^{\prime }(90^{\circ }))\\ 2/\sqrt{3}\times (I^{\prime }(45^{\circ })-I^{\prime }(90^{\circ }))\\ I_{r}^{\prime }-I_{l}^{\prime } \end{array}\right]$$ (4)

The change of polarization parameter of light can describe the change of polarization state of light well, where the polarization degree is one of the important parameters of the polarization parameter of light, the definition of polarization degree is the proportion of the polarized light component to the whole light wave component, divided into three kinds: Degree of Polarization (DOP), Degree of Linear Polarization (DOLP) and Degree of Circular Polarization (DOCP), their expressions are [22, 23]:

$$\mathrm{DOP}=\frac{\sqrt{Q^{2}+U^{2}+V^{2}}}{I}$$ (5)

$$\mathrm{DOLP}=\frac{\sqrt{Q^{2}+U^{2}}}{I}$$ (6)

$$\mathrm{DOCP}=|\frac{V}{I}|$$ (7)

Angle of Polarization (AOP) is also an important parameter in the polarization parameter, defined as the phase difference between the two radiation components, the expression is:

$$\mathrm{AOP}=\frac{1}{2}\arctan(\frac{U}{Q})$$ (8)

By using the above formula for the Stokes vector, the polarization parameters can be further derived, which results in the polarization information of the detection target.

## Multi-spectral polarization imaging detection scheme based on micro-polarizer array

### Micro-polarizer array design

**Fig 1:**
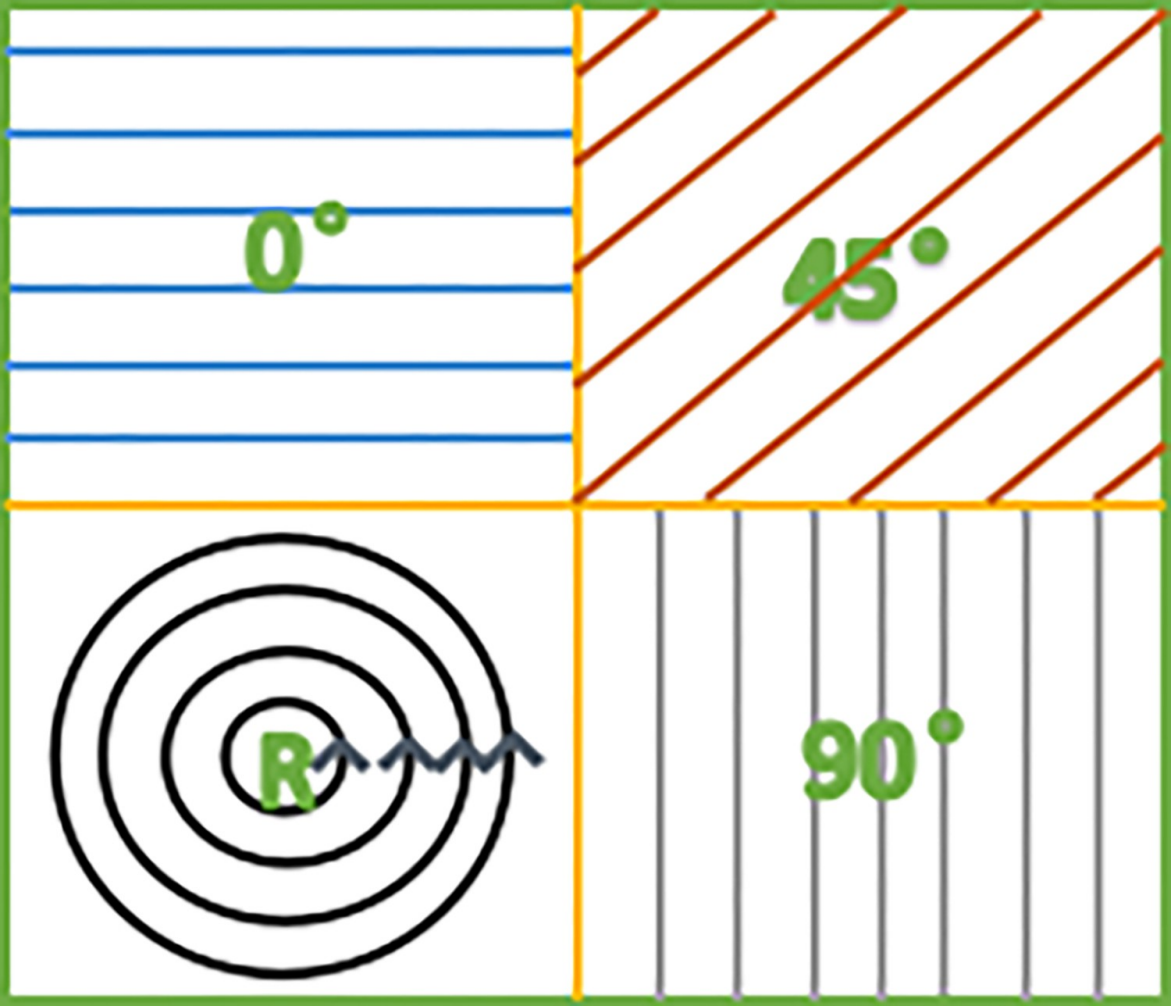
Image division design scheme. (the four regions of intensity are I(0°), I(45°), I(90°) and I(R)).

Micro-polarization imaging technology uses a Micro-polarizer Array (MPA) image sensor to capture images. The MPA is integrated on the photographic chip, and the cell size of the MPA is exactly the same as the pixel cell size of the photographic chip to be integrated, and the cells of the MPA are aligned one by one with the pixel cells of the photographic chip [24]. In this study, the detector target surface is divided into 2×2 areas, and the detected light intensity is the light intensity image after the detection of 0°, 45°, 90° and circular polarizer respectively. The advantage of this detector is that it can capture four different polarization direction images–horizontal, vertical, diagonal, and circular polarization–by capturing a single frame. As shown in Fig 1, the four regions of intensity are I(0°), I(45°), I(90°) and I(R).

**Fig 2:**
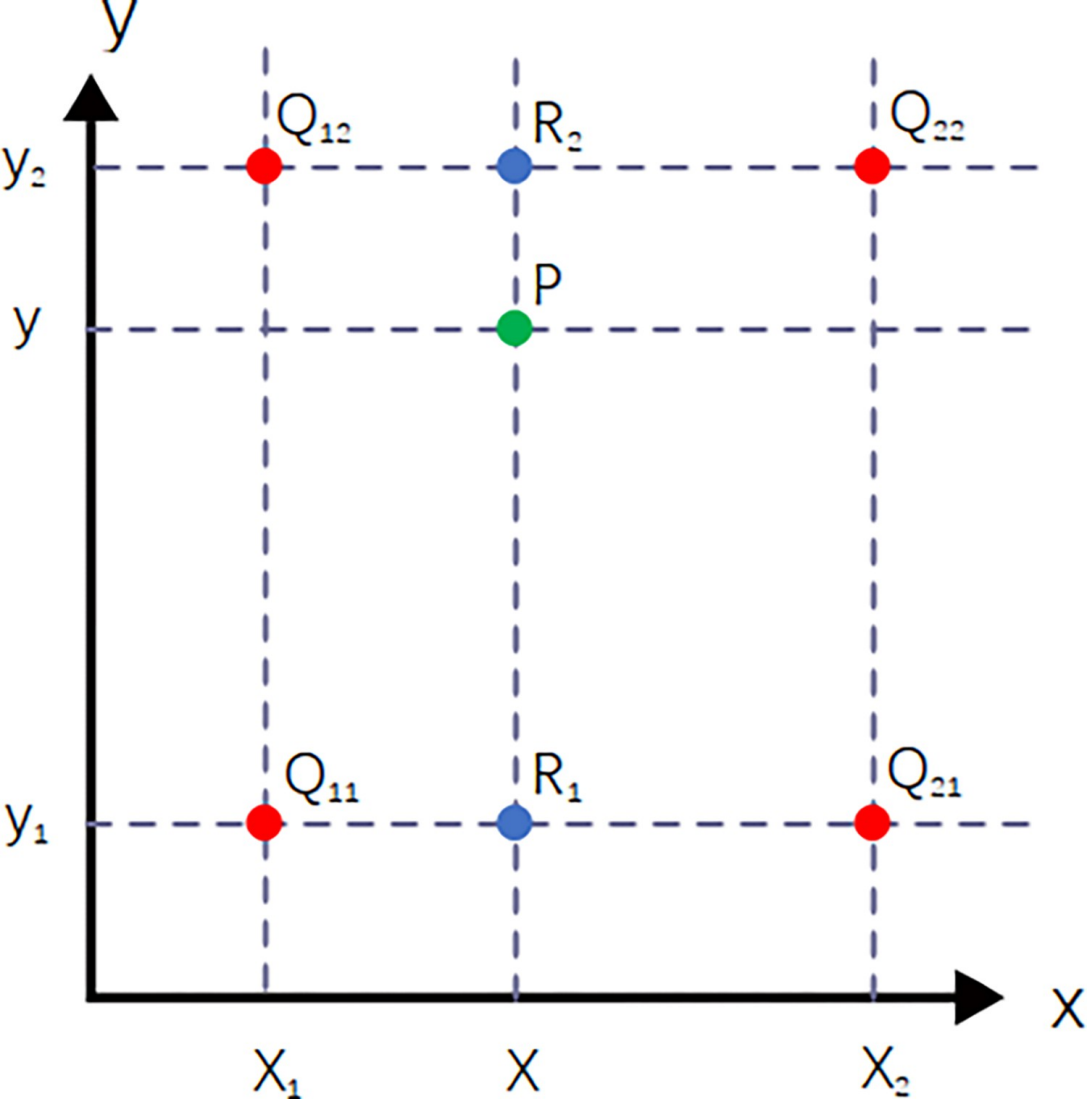
Schematic diagram of the bilinear interpolation method. (To get the gray value value of the unknown pixel point at the point P).

Every four image elements form a super image element. When obtaining a single frame of spatial target polarization image, it is necessary to extract the pixel units with the same polarization direction according to the arrangement of the micro-polarizer array and copy them into four different empty images. Since the image pixels with polarization spectrum multiparameter detection information are lost by half in the process of sub-regioning the pixels, the Interpolation between two adjacent pixels is used [25] to make up the number of pixels, and a bilinear interpolation method is used to make up the data of the null region between two adjacent pixels. The bilinear interpolation method is shown in Fig 2 To get the gray value value of the unknown pixel point at the point P = (x, y), it is necessary to estimate the gray value using the pixel points with known gray value using Q_11_ = (x_1_, y_1_), Q_12_ = (x_1_, y_2_), Q_21_ = (x_2_, y_1_) and Q_22_ = (x_2_, y_2_).

**Fig 3:**
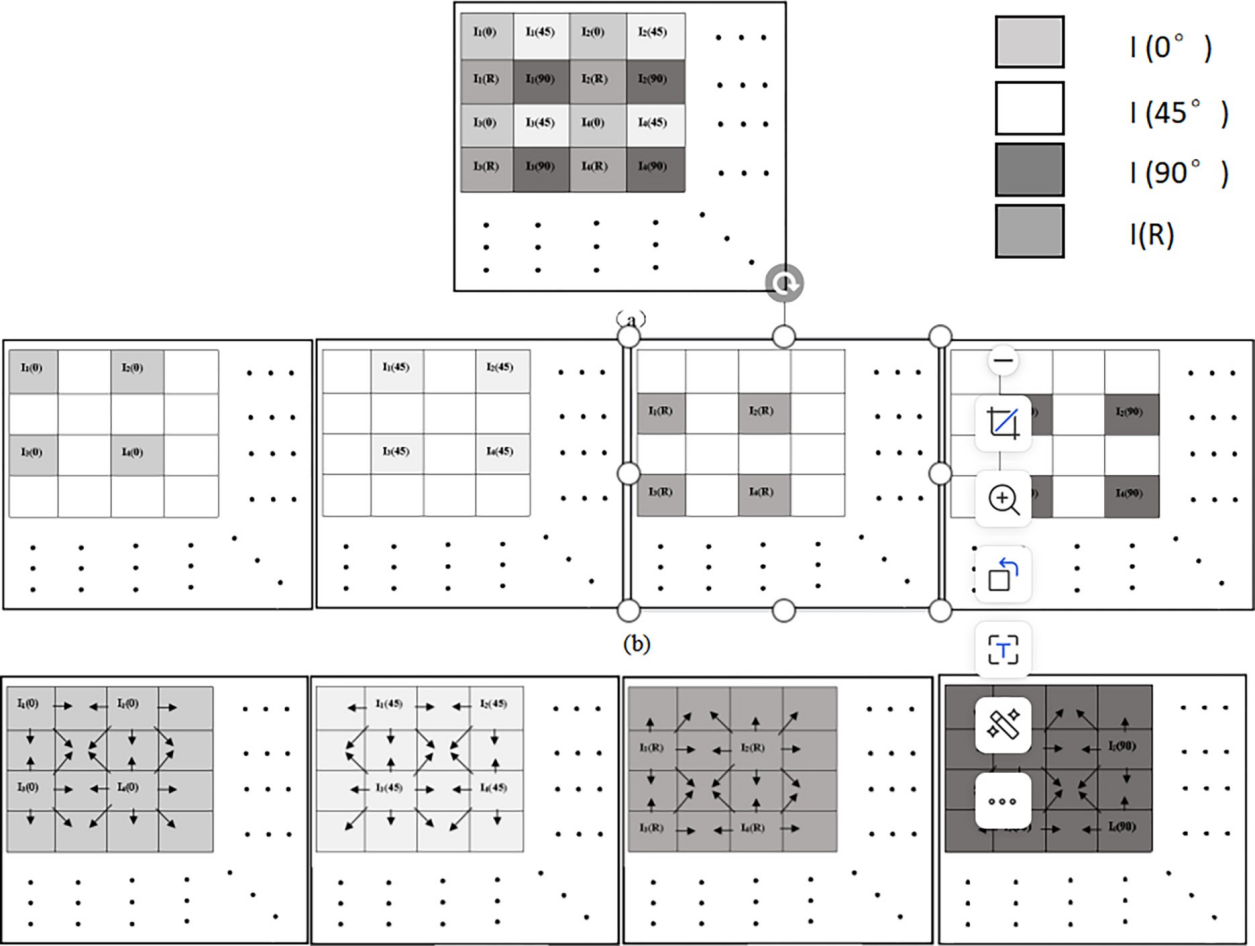
Image processing method based on bilinear interpolation.

Compared with other interpolation methods, the bilinear interpolation method has the advantages of simple algorithm structure and easy implementation, and the obtained images have high pixel resolution, which can highlight the target details and structure and other features in the images more obviously. Four images with different detection bias directions can be obtained with light intensity values of I(0°), I(45°), I(90°) and I(R), which contain 1/2 of the null region. By convolving 2×2 pixel cells on the array, the polarization state measurements at the vacant positions can be fitted to improve the spatial resolution of the data [26]. Finally, the real-time detection of four Stokes polarization spectrum multiparametric detection parameters is achieved and the pixel size requirement is satisfied. After obtaining the polarization images with four different polarization directions by using the bilinear interpolation method, by combining the Stokes formula, spatial polarization degree image and polarization angle image of the spatial target can be obtained. The image processing method based on bilinear interpolation is shown in Fig 3.

**Fig 4:**
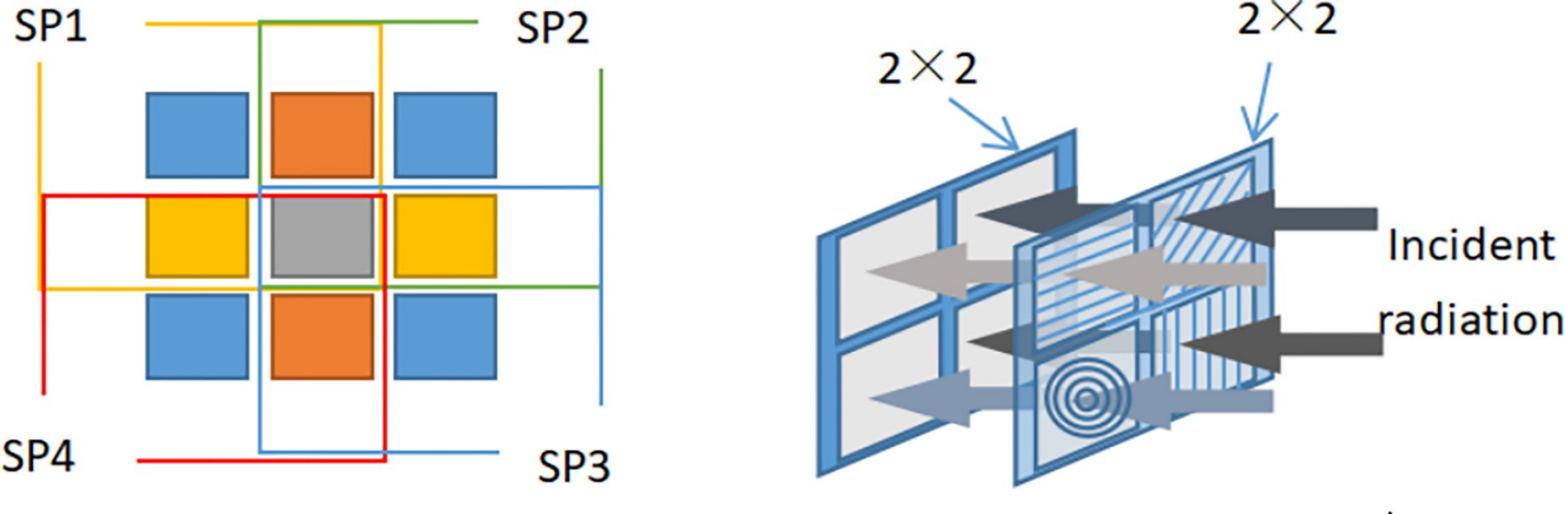
Schematic diagram of micro-polarizer split-focal plane polarization imaging. ((a)Micropolarizer array distribution model (b)The micropolarizer array corresponds to the photosensitive chip one by one).

This paper is a micro-polarizer array designed based on the fractional plane polarization imaging technique. Compared with the traditional fractional plane polarization imaging technique, its advantage lies in the ability to obtain micro-polarizer arrays with good performance and easy preparation of full Stokes vectors. Based on the in-depth study of the polarization selective transmission mechanism of the micro-nano grating, the structure and corresponding parameters of the micro-nano grating to obtain the full Stokes vector are optimized and designed to provide theoretical and technical support for the focal plane full polarization detection technique. The design idea is to improve the polarization selective transmission performance (high polarization transmittance, extinction ratio and wide spectral bandwidth) in the visible wavelength range by optimizing the structural parameters based on the study of polarization selective transmission characteristics of micro-nano wire grating and micro-nano circular grating. The schematic diagram of the micro-polarizer split-plane polarization imaging is shown in Fig 4.

In this paper, the micro-polarizer manufacturing technology based on metal micro-nano grating, which has the advantages of minimal inter-pixel crosstalk, thinner thickness, and stable polarization performance, good polarization effect and high polarization efficiency.

In addition to the manufacturing process which affects the performance of the micro-polarizer array, each design parameter of the micro-polarizer array: substrate material, grating period, grating width, grating slot depth, etc. also affects the performance of the micro-polarizer array and thus the imaging quality, so these parameters need to be designed.

Wire grids composed of low refractive index have better transmittance and extinction ratio than high refractive index. When the transmittance of the base material is comparable in a specified wavelength range, it is advisable to choose a material with a low refractive index. We choose a high transmittance glass with 92% transmittance and 1.47 refractive index as the substrate material.

When the grating period is much smaller than the incident light wavelength, the grating has only zero-level diffraction waves, at this time the grating can be used as a good polarization device, so the grating needs to meet the zero-level diffraction conditions [27]:

$$T(n\sin\theta_{m}+n_{i}\sin\theta_{i})=m\lambda$$ (9)

Where *T* is the grating period, *m* is the diffraction level, *n* is the refractive index of the substrate, *θ*_*m*_ is the corresponding diffraction angle, *n*_*i*_ is the refractive index of air, and *θ*_*i*_ is the angle of incidence. Because there is only zero diffraction, so *m* = 1, *θ*_*m*_ = *π*/2, the refractive index of the substrate *n* = 1.47, when the incident angle is 0, the grating period should be less than the critical period value of 160nm. When the duty cycle is set to 0.6, and the grating period ranges from 0 to 200nm, the main transmittance with wavelength and period variation is calculated using Eqs (10) to (12) [27].

$$T_{TM}=\frac{4nA^{2}}{1+{\left(1+n\right)}^{2}A^{2}}$$ (10)

$$\begin{array}{r} \frac{1}{A}=\frac{4T}{\lambda }\{\ln[\csc\frac{\pi (T-a)}{2T}]+\frac{Q\cos^{4}[\pi (T-a)/2T]}{1+Q\sin^{4}[\pi (T-a)/2a]}+\\ \frac{1}{16}{\left(\frac{T}{\lambda }\right)}^{2}{\left[1-3\sin^{2}\frac{\pi (T-a)}{2T}\right]}^{2}\cos^{4}\frac{\pi (T-a)}{2T}\} \end{array}$$ (11)

$$Q=\frac{1}{{\left[1-{\left(T/\lambda \right)}^{2}\right]}^{1/2}}-1$$ (12)

**Fig 5:**
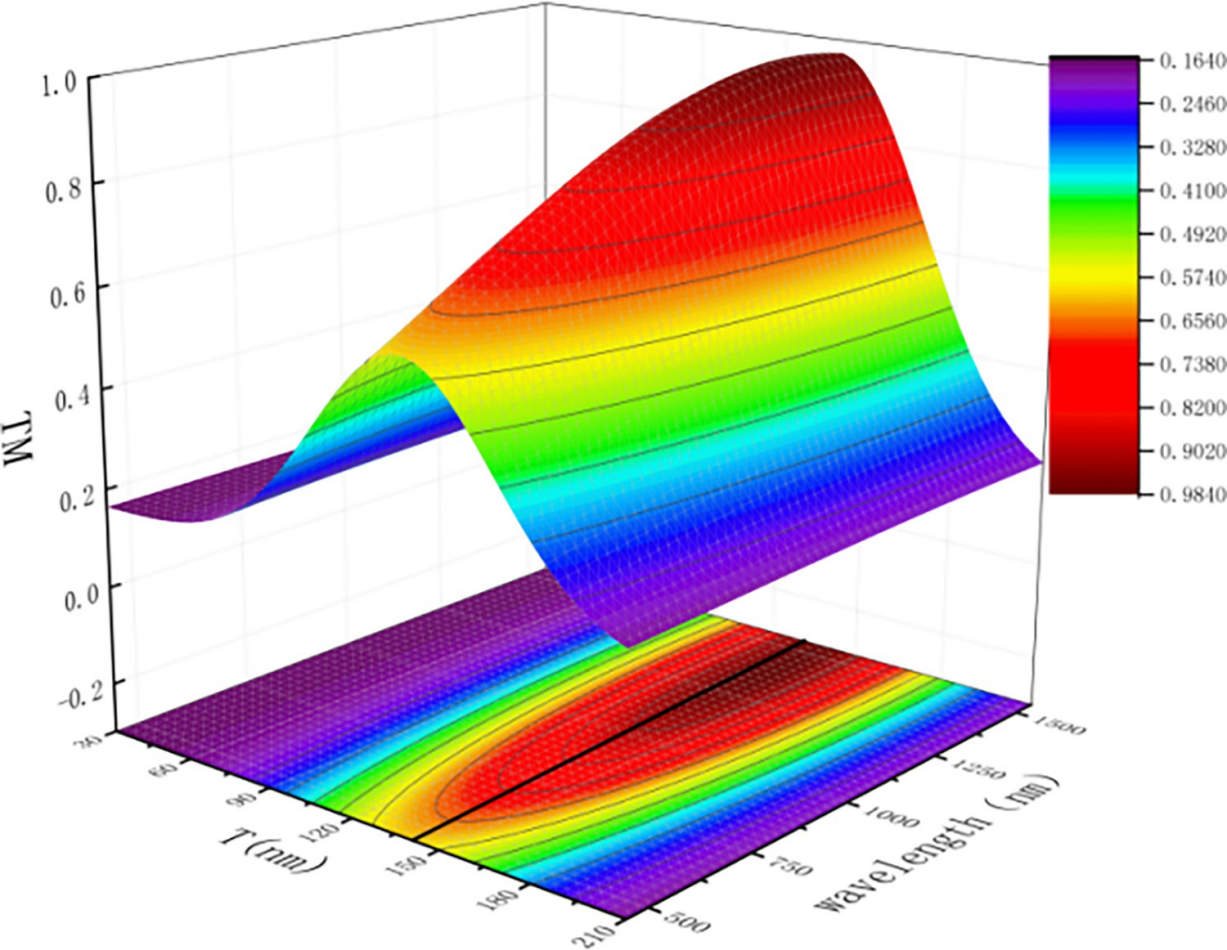
The effect of grating period on TM transmittance. (simulated picture).

In these equations, *T* represents the grating period, *A* represents the grating width, and *λ* represents the wavelength of the incident light. The simulation results are shown in Fig 5.

From Fig 5., it can be observed that as the wavelength increases, the main transmittance gradually increases. At any given wavelength, the main transmittance shows a trend of initially increasing and then decreasing with an increase in the grating period. When the grating period is 140nm, the TM transmittance is the highest. Therefore, the grating period is set to 140nm.

**Fig 6:**
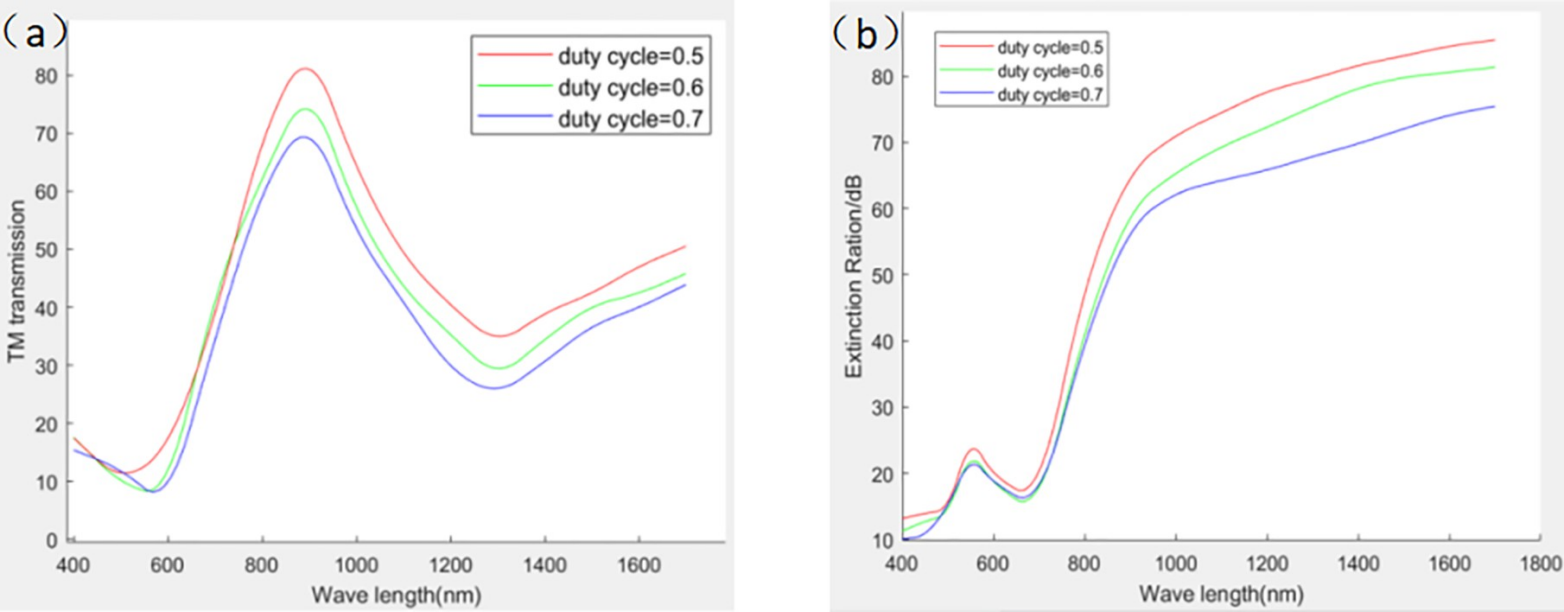
Effect of duty cycle on TM transmittance and extinction ratio. ((a) The effect of duty cycle on TM transmittance (b) The effect of duty cycle on extinction ratio)).

The grating duty cycle is defined as the ratio of the wire grid width to the grating period, and the TM wave transmittance and extinction ratio are traded off by varying the grating duty cycle. As the duty cycle increases, the distance between the grating and the grating decreases, resulting in a decrease in TM wave transmission. However, in this process, the TM wave attenuation is relatively small, which leads to an increase in the extinction ratio. Therefore, considering the effect of the grating duty cycle on the TM wave transmittance and extinction ratio, the TM wave transmittance and extinction ratio can reach a high level when the duty cycle is f = 0.5, as shown in Fig 6.

**Fig 7:**
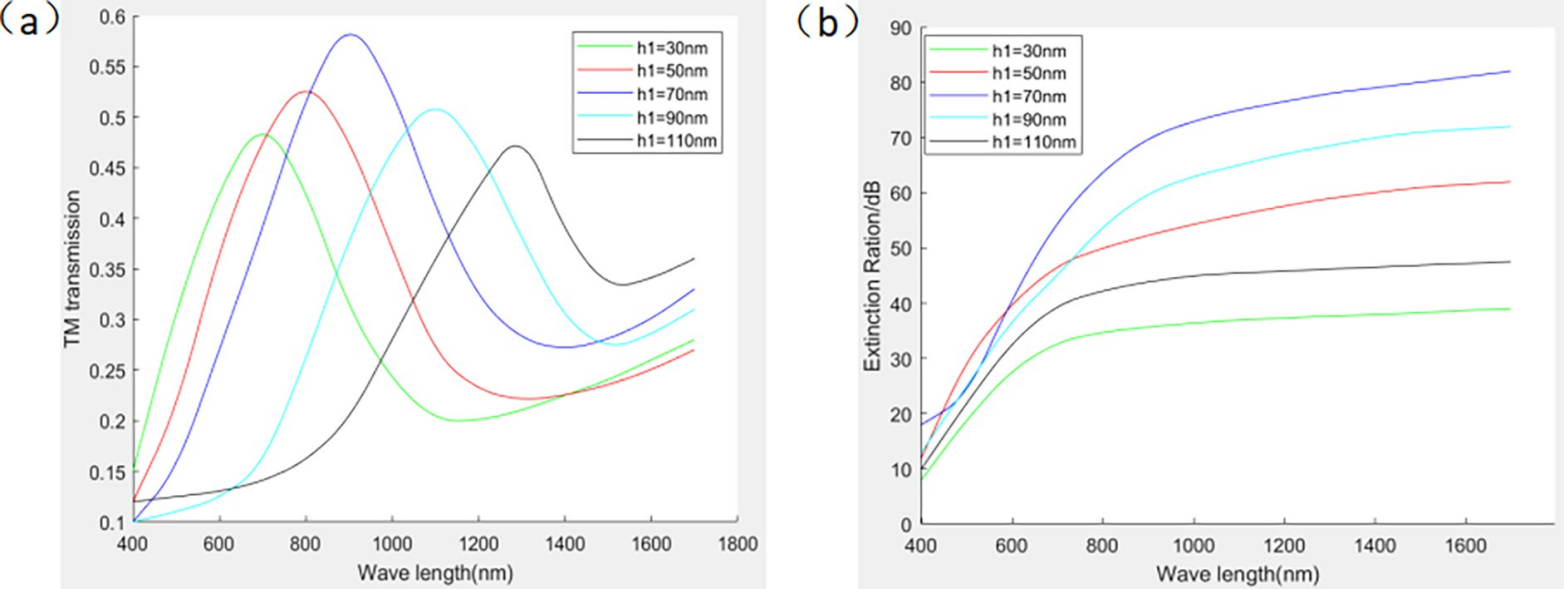
Effect of grating slot depth on TM transmittance and extinction ratio. ((a) Effect of grating slot depth on TM transmittance(b) Effect of grating slot depth on extinction ratio).

The depth of the grating groove is also a key parameter for the polarization performance of metal gratings, and the aspect ratio is the ratio of the grating groove depth to the width of the wire grating. The relationship between grating depth and grating polarization performance is shown in Fig 7 When the depth of the aluminum layer *h*_1_ is chosen to be 70 nm, the TM transmittance and extinction ratio are at a high level. Since the design is a double-layer grating, the sum of the aluminum layer depth *h*_1_ and the highly transmissive glass layer depth *h*_2_ should be 140*mm*. Therefore, the depth of the highly transmissive glass layer *h*_2_ is 70 nm.

According to the imaging method of micro-polarization real-time imaging, the field of view of each image element is different, so the Instantaneous Field of View (IFOV) of adjacent pixels does not overlap in principle, and the direct calculation of polarization information will cause an alignment error of one pixel. Therefore, it is necessary to analyze and correct the instantaneous field of view error.

In this section, a method is proposed to estimate the interpolation error by combining Newton polynomial interpolation and polarization difference model, and incorporating an edge classifier in the polarization difference domain. This method aims to reduce interpolation errors caused by high-frequency information such as object edges during reconstruction, which is often overlooked in traditional polynomial interpolation methods. The equation of this algorithm is obtained using Newton’s polynomial and Taylor’s formula as follows [28]:

$${\tilde{I}}_{90}(i,j-1)≅\frac{I_{90}(i,j-2)+I_{90}(i,j)}{2}-\frac{I_{135}(i,j+1)-2I_{135}(i,j-1)+I_{135}(i,j-3)}{8}$$ (13)

The interpolation predictor for the 45° diagonal direction in the polarization difference domain is defined as

$${\hat{I}}_{0}^{45^{o}}(i,j)=I_{90}(i,j)+\frac{I_{0}(i+1,j-1)+I_{0}(i-1,j+1)}{2}-\frac{\tilde{I_{90}}(i+1,j-1)+\tilde{I_{90}}(i-1,j+1)}{2}$$ (14)

The interpolation predictor for the -45° diagonal direction in the polarization difference domain is defined as

$${\hat{I}}_{0}^{-45^{o}}(i,j)=I_{90}(i,j)+\frac{I_{0}(i-1,j-1)+I_{0}(i+1,j+1)}{2}-\frac{\tilde{I_{90}}(i-1,j-1)+\tilde{I_{90}}(i+1,j+1)}{2}$$ (15)

Let $\varphi^{45^{o}}$ and $\varphi^{-45^{o}}$ be the loss values in the 45° and -45° diagonal directions, respectively, defined as

$$\begin{array}{l} \varphi^{45^{o}}=\sum_{m=\{-2,0,2\}}\sum_{n=\{-2,0,2\}}\left|{\hat{I}}_{0}^{45^{o}}(i+m,j+n)-I_{90}(i+m,j+n)\right|\\ \varphi^{-45^{o}}=\sum_{m=\{-2,0,2\}}\sum_{n=\{-2,0,2\}}\left|{\hat{I}}_{0}^{-45^{o}}(i+m,j+n)-I_{90}(i+m,j+n)\right| \end{array}$$ (16)

The ratio of the two loss values is used as the edge classifier and is calculated as follows:

$$\Phi =\max(\frac{\varphi^{45^{o}}}{\varphi^{-45^{o}}},\frac{\varphi^{-45^{o}}}{\varphi^{45^{o}}})$$ (17)

**Fig 8:**
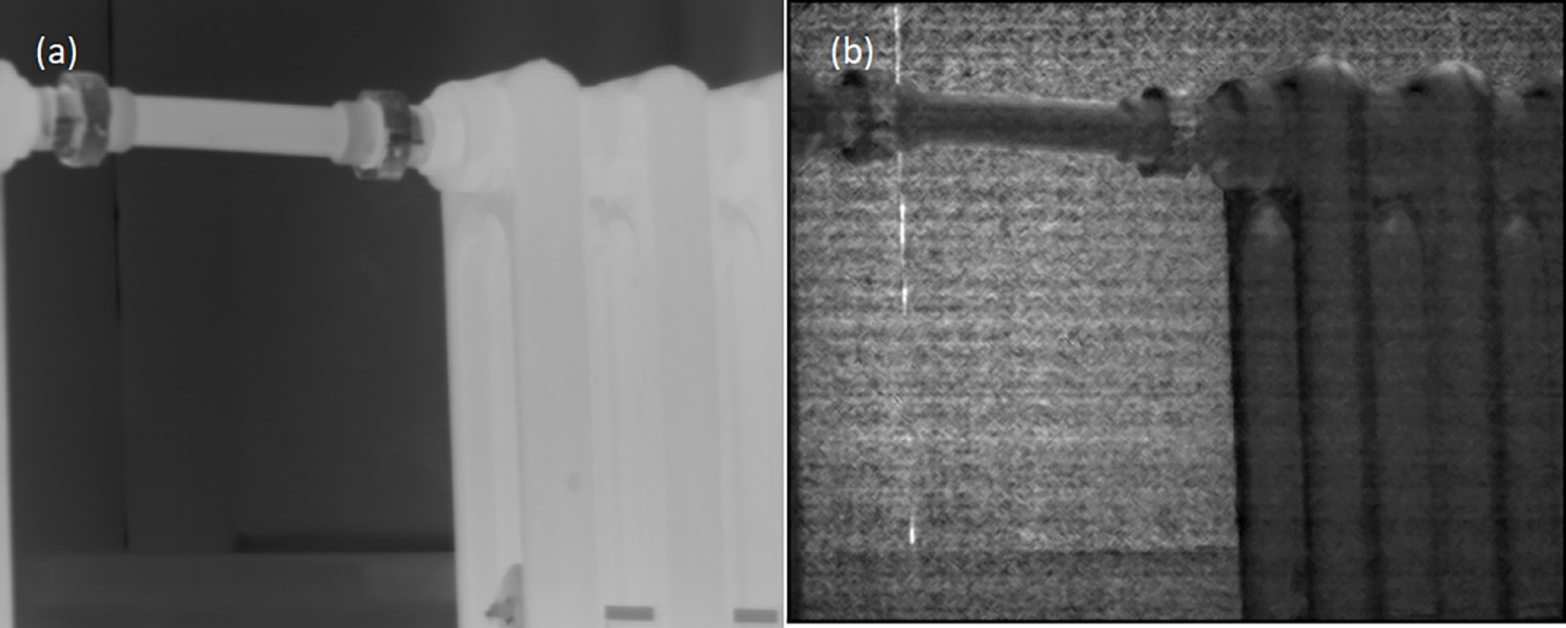
Target polarization map using Newton’s interpolation algorithm. ((a) Original image(b) Newton polynomial interpolation).

Fig 8 shows the image data acquired by an infrared polarization imaging system using an array of micro-polarizers. Fig 8(A) shows the original image and Fig 8(B) shows the polarization degree image obtained after processing by the Newton polynomial interpolation algorithm. Due to the characteristics of the micro-polarization real-time imaging method, there will be transient field-of-view error, which leads to jagged false information in the image and affects the distinction of target details. Using the Newton polynomial interpolation algorithm, it can be seen that the target edges are almost jagged-free and the target surface details are displayed more clearly.

## Multi-spectral polarization imaging detection device

**Fig 9:**
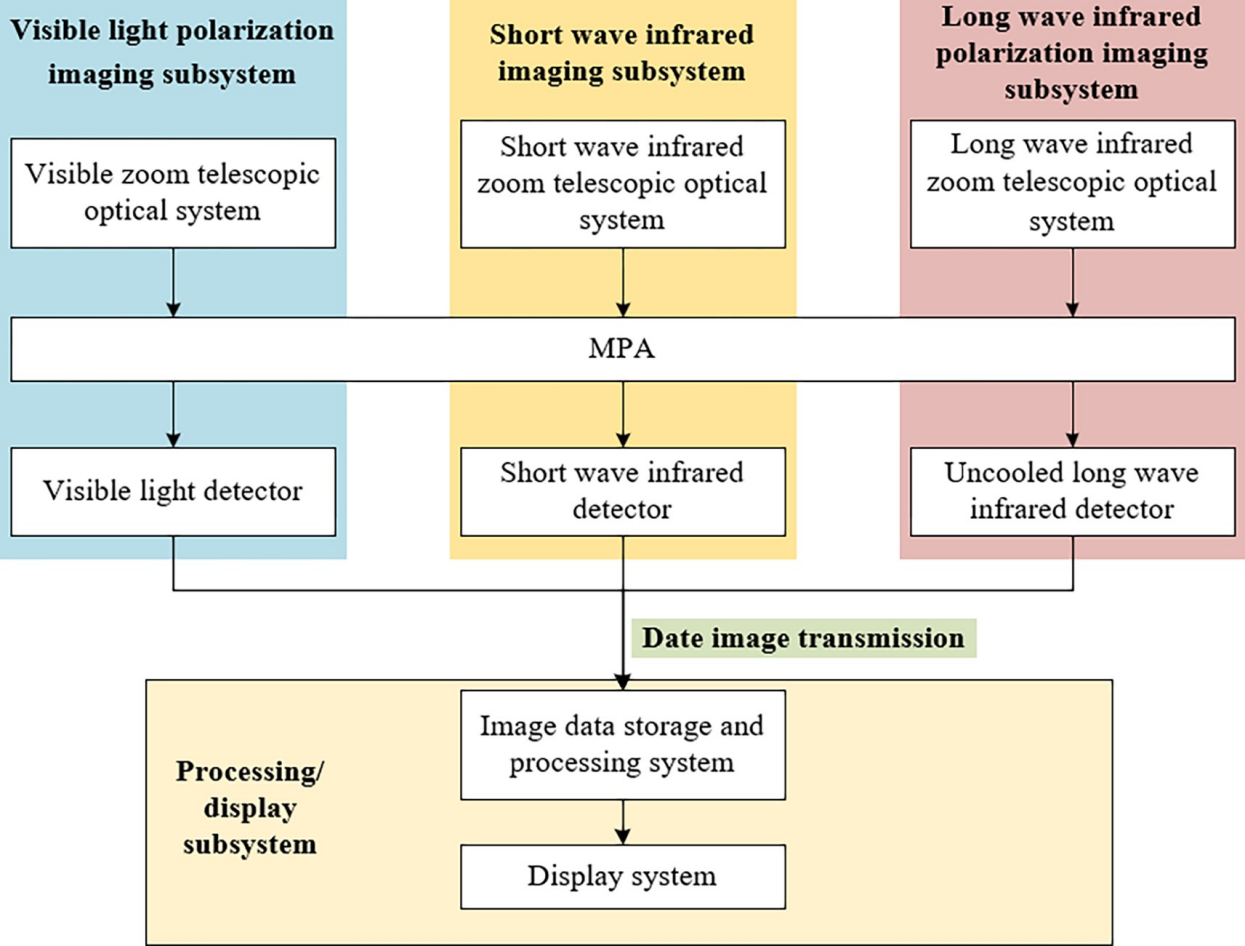
Block diagram of system composition.

**Fig 10:**
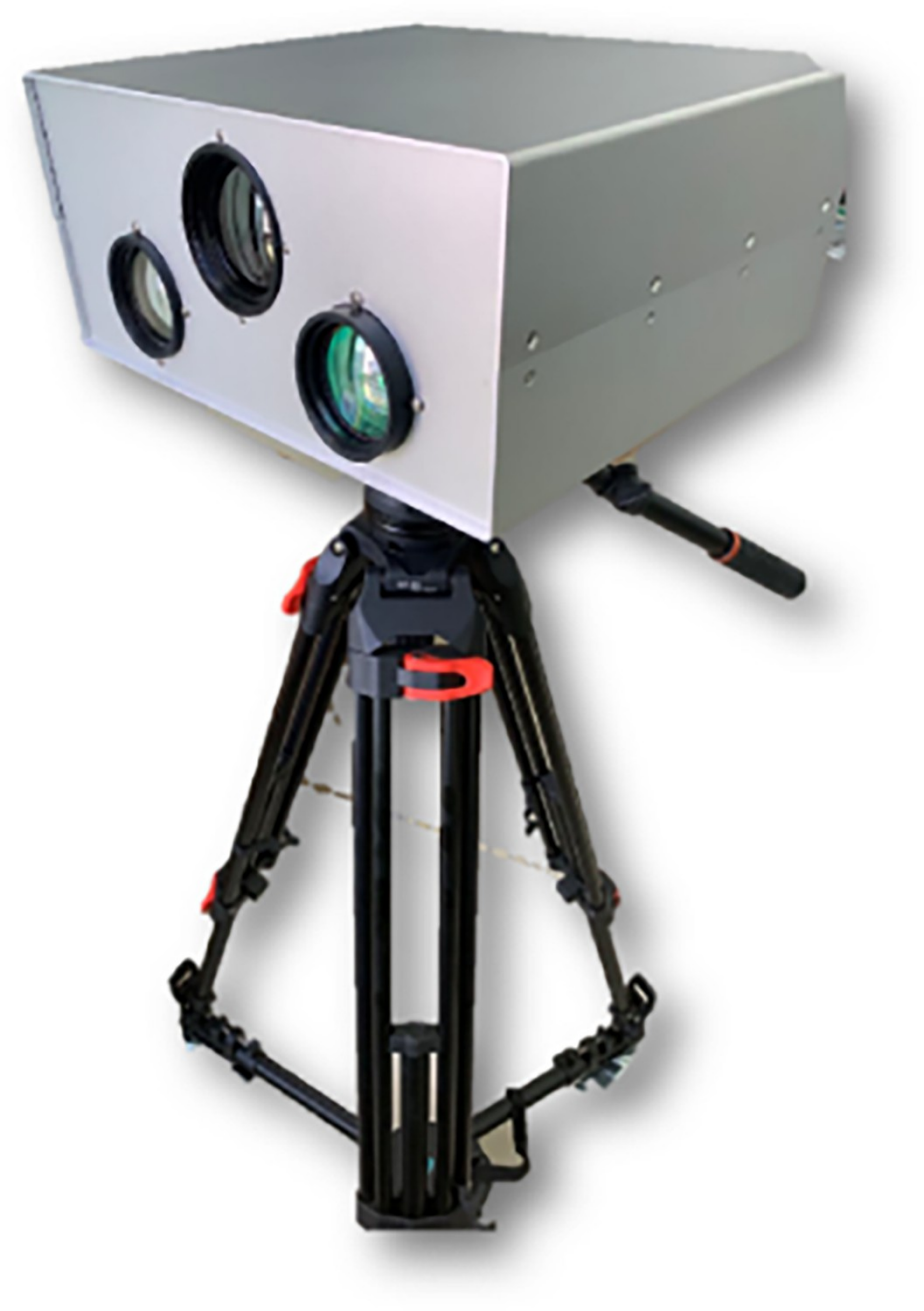
Physical diagram of multispectral polarization detection.

The multispectral polarization imaging detection system consists of four parts: visible polarization imaging subsystem, short-wave infrared imaging subsystem, long-wave infrared polarization imaging subsystem, and processing/display subsystem. The system composition is shown in Fig 9 below. The multispectral polarization imaging detection device is shown in Fig 10.

Functions of the components:

1. visible polarization imaging subsystem: the visible zoom telescope optical system to obtain the target visible band (0.4 ~ 0.8um) information, after polarization spectral components will be obtained respectively 0°, 45°, 90° and R four angle polarization light intensity, the use of visible light detector for reception.
2. short-wave infrared imaging subsystem: the short-wave infrared zoom telescope optical system to obtain the target short-wave infrared band (0.9~1.7um) information, using the short-wave infrared detector to receive.
3. Long-wave infrared polarization imaging subsystem: the long-wave infrared zoom telescope system to obtain the target long-wave infrared (8 ~ 12um) information, after polarization spectral components will be obtained respectively 0°, 45°, 90° and R four angle polarization light intensity, the use of long-wave infrared detector for reception.
4. Processing/display subsystem: collect and store the three waveband images, and fuse and enhance the images, and display them in real time through the monitor.

## Polarization imaging detection experiments

### Polarization imaging experiment in indoor simulation environment

**Fig 11:**
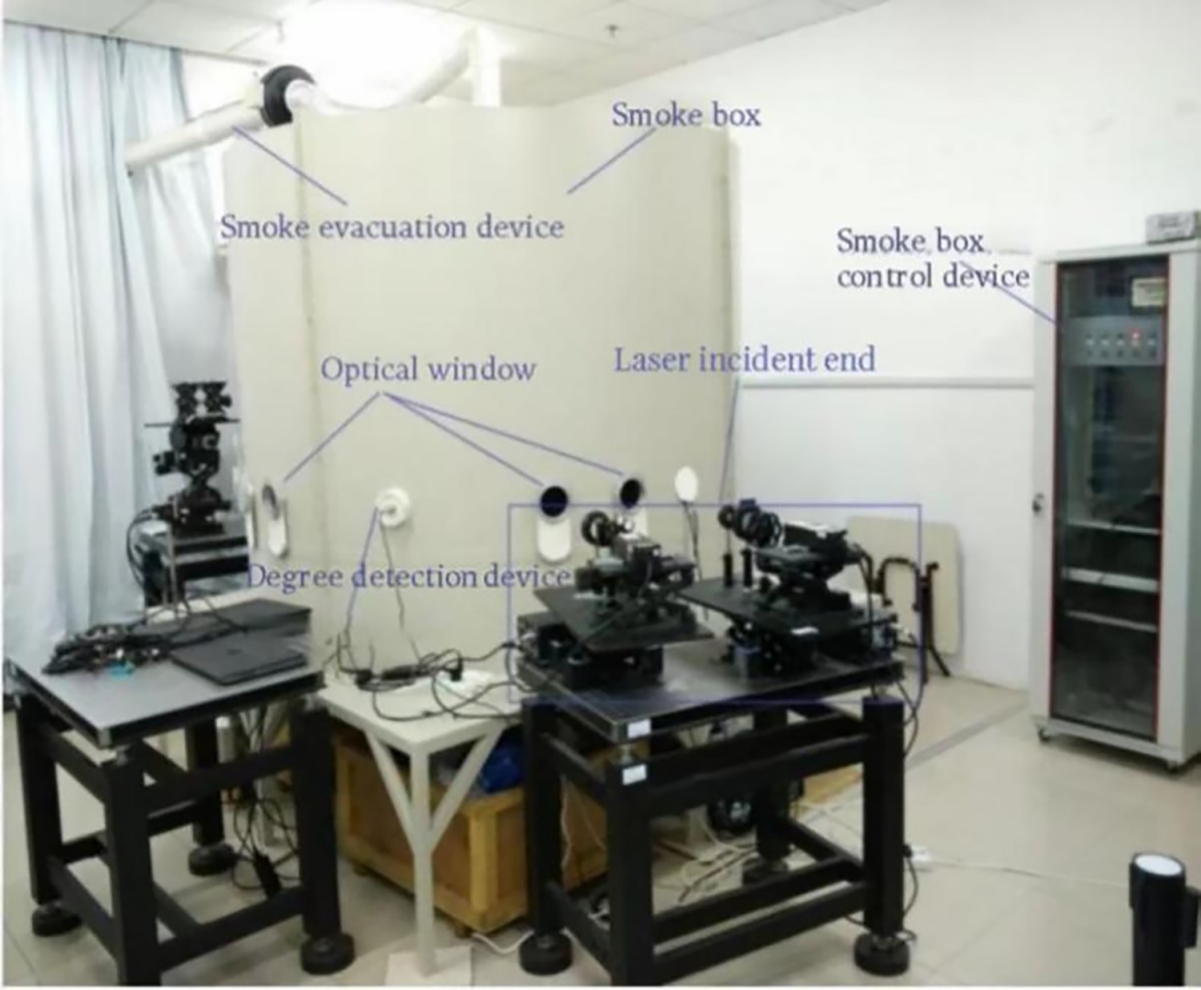
Physical view of polarization imaging in smoke simulation environment.

We simulated the smoke environment to verify the imaging effect of the designed micro-polarizer array. Fig 11 shows the field pictures of the polarization imaging experiment in the simulated indoor environment.

The polarization imaging experiment was conducted in a smoky environment with a transmittance of 71.4%, using a green cart and a white metal can as the target. To reduce the effect of light from other wavelengths, a 530 nm filter (which filters out light of colors other than green) was added in front of the lens for the experiment.

**Fig 12:**
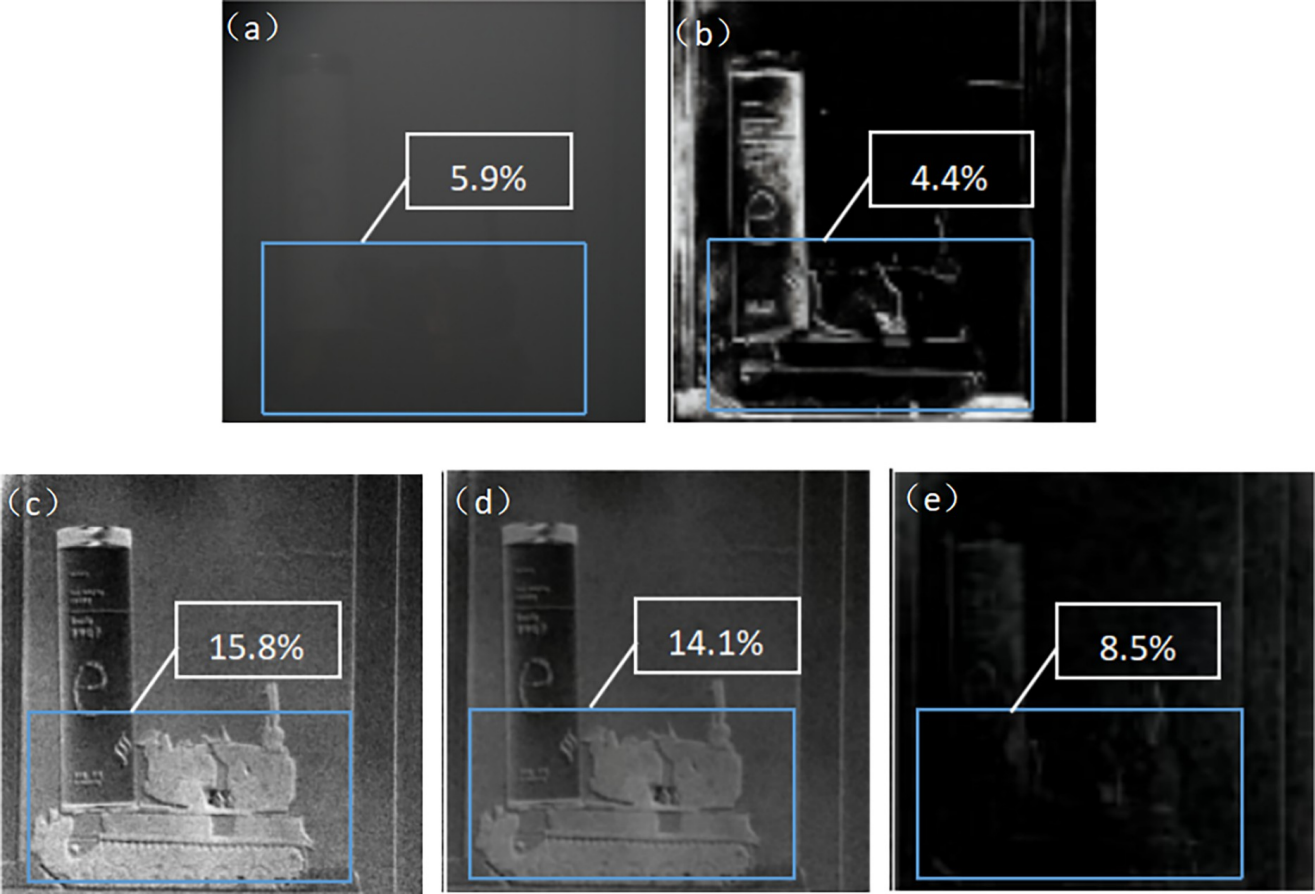
Target image without MPA in visible smoke environment. ((a)Intensity images(b) V image(c) DOP image(d) DOCP image(e) AOP image).

First, use a regular intensity camera to obtain the intensity image of the target. Then, use a regular polarized camera to image the target, capturing intensity images at 0°, 45°, and 90° respectively. Finally, apply Eqs (4) to (8) to generate the V, DOP, DOCP, and AOP images (Fig 12).

**Fig 13:**
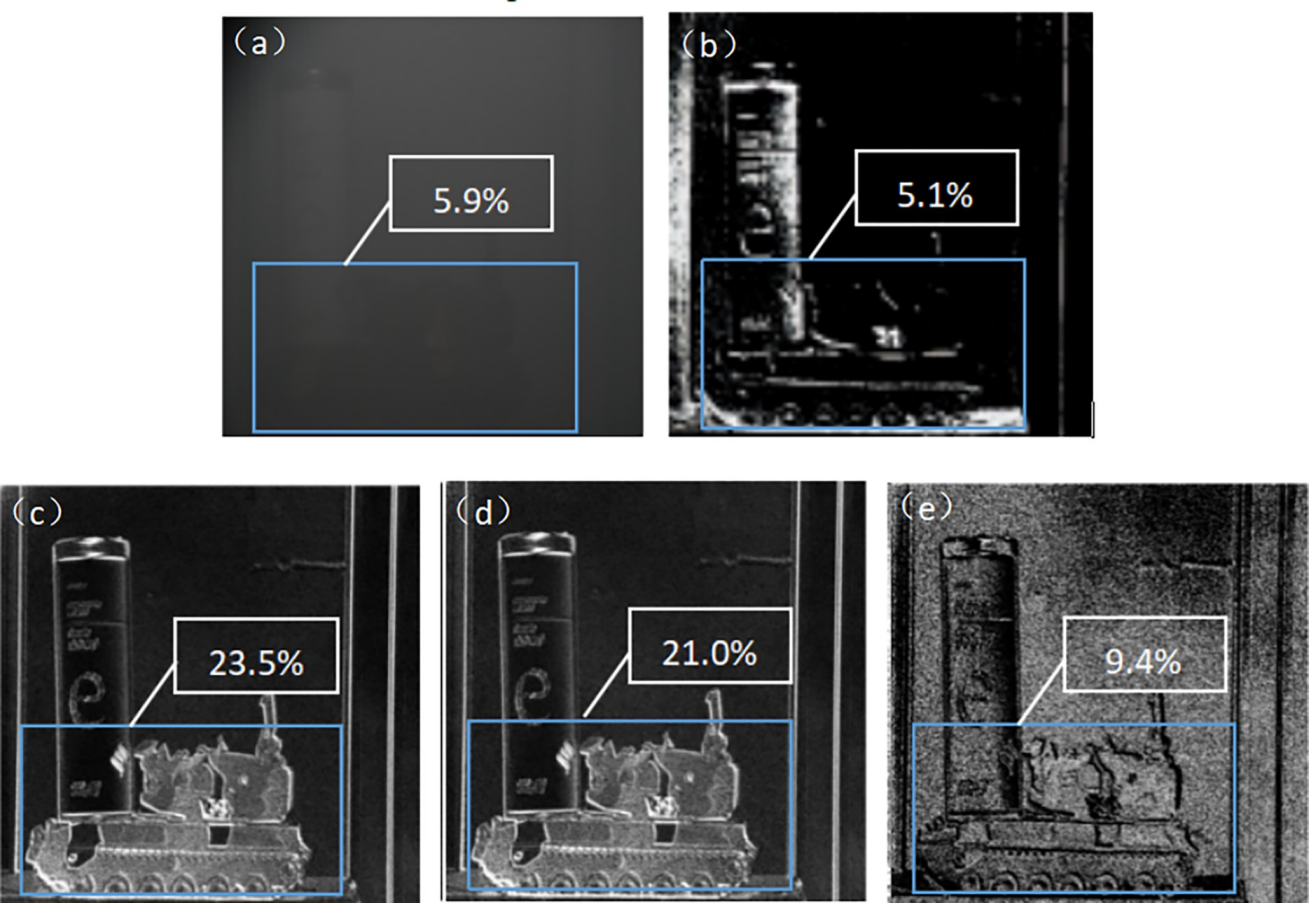
Target images obtained using a haze transmission polarization imaging detection system in a visible smoke environment. ((a)Intensity images(b) V image(c) DOP image(d) DOCP image(e) AOP image).

Using the designed haze-polarized imaging detection system (employing MPA), we obtained intensity images of the target at 0°, 45°, and 90° angles. By applying Eqs (4) to (8), we can calculate V, DOP, DOCP, and AOP images (Fig 13).

By comparing Figs 12 and 13, it can be observed that the polarization imaging detection system using the designed MPA for haze penetration exhibits higher contrast and clearer images of the target under the same smoky conditions. In Fig 13, (a) is an intensity image that mainly focuses on the brightness information, with a contrast of 5.9%. The contrast of the DOP image is 23.5%, the contrast of the DOCP image is 21.0%, and the contrast of the AOP image is 9.4%. All of these contrast values are higher than the intensity image, enabling the enhancement of the target’s edge contours and making the target easier to identify. In the AOP image, in addition to the polarization angle information, it is also noticeable that there is a significant amount of noise. This is because the polarization angle ranges from −*π*/2 to *π*/2, corresponding to colors from black to white. If the angle exceeds *π*/2, the color transitions from white to black, and if it falls below -X, the color transitions from black to white. It is precisely due to these transitions that the AOP image exhibits noise, but its contrast is still higher compared to the intensity image. Therefore, in smoky environments, polarization imaging technology has certain advantages in highlighting the edge contours of the target.

The image contrast is calculated by the formula:

$$C_{W}=\frac{I_{t}-I_{b}}{I_{b}}$$ (18)

Where the target background contrast of the intensity image *C*_*W*_, *I*_*t*_ indicates the target area brightness, *I*_*b*_ is the overall brightness of the background area of the image.

**Fig 14:**
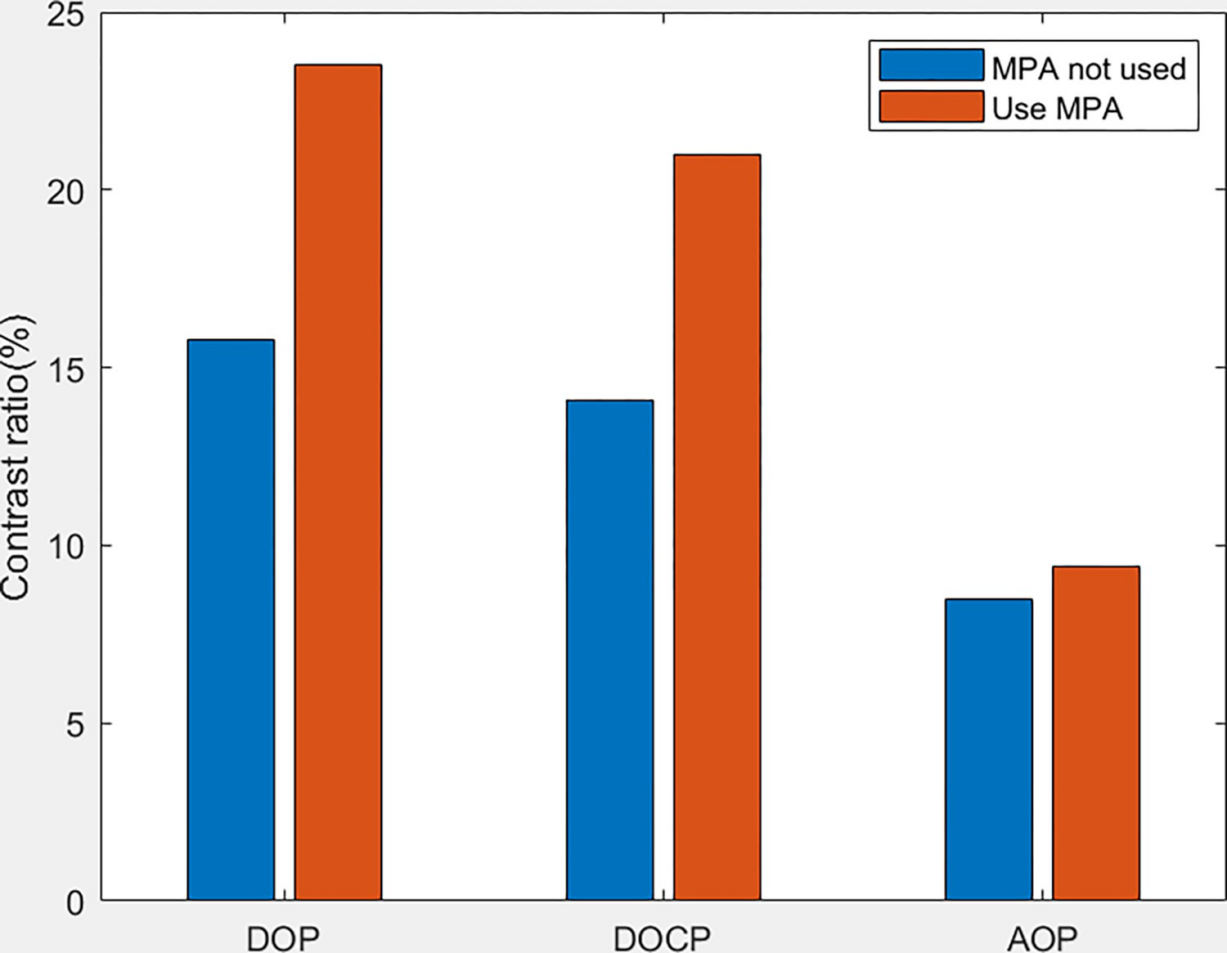
Comparison of contrast between target image with MPA and without MPA.

Based on Fig 14, it can be observed that the imaging system with MPA provides better polarization imaging results compared to the system without MPA. The contrast of DOP, DOCP, and AOP is all improved when MPA is incorporated. Additionally, the imaging system with MPA exhibits the highest contrast in the DOP image.

### Polarization imaging experiment in real outdoor environment

Test experiments on polarized light transmission imaging under different haze conditions were carried out. The following figure shows the test results of the haze transmission multi-spectral polarization imaging detection system with an ordinary intensity camera and using the MPA designed in this paper in the presence of fog.

**Fig 15:**
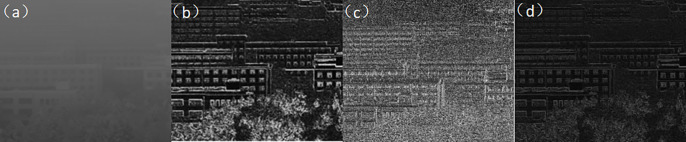
Comparison of unbiased external field and polarization experimental images in the visible range under foggy weather conditions. ((a) Normal camera intensity(b) DOP(c) AOP(d) DOCP).

**Fig 16:**
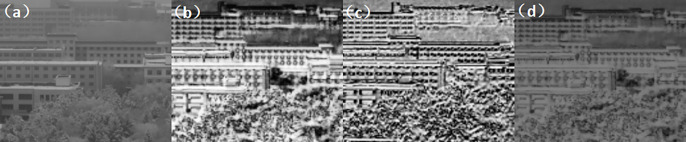
Comparison of unbiased external field and polarization experimental images in the short-wave infrared range under foggy weather conditions. ((a) Normal camera intensity(b) DOP(c) AOP(d) DOCP).

**Fig 17:**
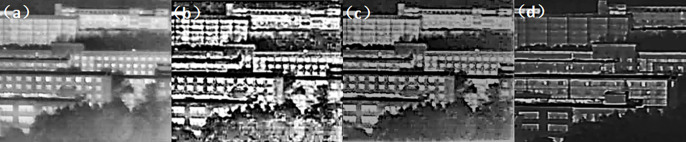
Comparison of unbiased external field and polarization experimental images in the long-wave infrared range with foggy weather conditions. ((a) Normal camera intensity(b) DOP(c) AOP(d) DOCP).

Experiment 1: With an atmospheric visibility of 1 km and a detection distance of 1 km, tests were conducted on three different wavelength bands: visible light, short-wave infrared, and long-wave infrared. After comparing Figs 15–17, the results show that polarization imaging detection can highlight target edges and improve imaging quality compared to regular intensity images.

**Fig 18:**
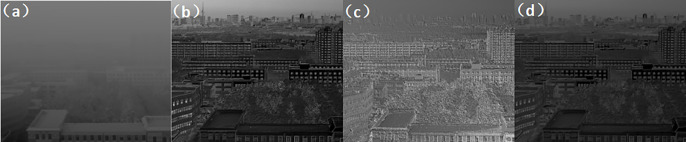
Comparison of visible light unbiased external field and polarization experimental images in foggy weather condition. ((a) Normal camera intensity(b) DOP(c) AOP(d) DOCP).

**Fig 19:**
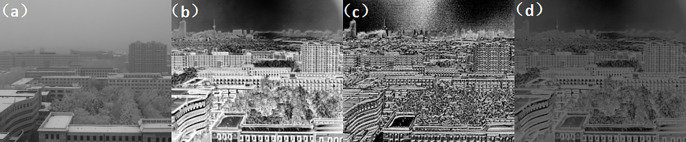
Comparison of short-wave infrared unbiased external field and polarization experimental images under foggy weather conditions. ((a) Normal camera intensity(b) DOP(c) AOP(d) DOCP).

**Fig 20:**
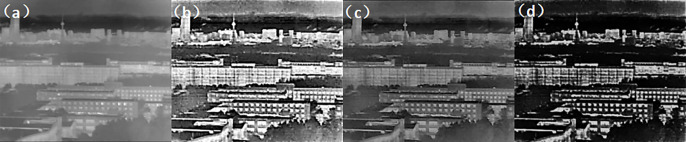
Comparison of long-wave infrared unbiased external field and polarization experimental images under foggy weather conditions. ((a) Normal camera intensity(b) DOP(c) AOP(d) DOCP).

Second experiment: atmospheric visibility of 1km, detection range of 5km. (Figs 18–20)

From the above imaging results, it can be observed that with a detection range of 1km, a regular strength camera is unable to provide clear imaging of the buildings at that distance. Similarly, with a detection range of 5km, the regular strength camera is unable to detect the tower at that distance. However, by using an MPA imaging system with haze and multi-band polarization imaging capabilities, clear imaging at 1km and target recognition at 5km can be achieved, greatly enhancing the detection range.

In addition to this, the information entropy, mean gradient and standard deviation are calculated to objectively evaluate the polarized images of the target at different bands in the two sets of experiments.

The expression for the information entropy of an image is as follows [29]:

$$EN=-\sum_{i=0}^{L}p_{i}\log_{2}^{p_{i}}$$ (19)

Where, *L* is the total number of gray levels; *p*_*i*_ indicates the ratio between the number of pixels with a gray value of *i* and the total number of pixels. The EN value can be used to measure the amount of information contained in the image; the larger the EN value, the better the image quality and the richer the amount of information contained.

The average gradient is defined by the following equation [30], where *M*×*N* is the image size and *P*(*i*,*j*) is the pixel value at (*i*,*j*).

$$AG=\frac{1}{\left(M-1)(N-1\right)}{\sum_{i=1}^{M-1}\sum_{j=1}^{N-1}\left[\frac{{\left[F(i+1,j)-F(i,j)\right]}^{2}+{\left[F(i,j-1)-F(i,j)\right]}^{2}}{2}\right]}^{\frac{1}{2}}$$ (20)

The standard deviation is expressed as follows [31]:

$$STD=\sqrt{\frac{\sum_{i=1}^{M}\sum_{j=1}^{N}{\left(P(i,j)-\bar{P}\right)}^{2}}{M\times N}}$$ (21)

Where, $\bar{P}$ is the average of the image grayscale and the expression is as follows

$$\bar{P}=\frac{1}{M\times N}\sum_{i=1}^{M}\sum_{j=1}^{N}P(i,j)$$ (22)

The values of EN, AG, STD of target polarization imaging under different wavelengths in the first and second group of experiments calculated by Eqs (19)~(22) are shown in Tables 1–4. Comparing the data in the tables, it can be seen that the multi-spectral polarization imaging detection system we designed for haze transmission can improve the imaging quality at different detection distances.

**Table 1:** Evaluation indexes of intensity camera target images in the first group of experiments.

	*EN*	*AG*	*STD*
Visible light	5.4086	1.3845	12.6645
Short-wave infrared	6.0228	5.6561	18.3213
Long-wave infrared	7.6094	15.2762	50.5361

**Table 2:** Evaluation indexes of DOP images of different wavelengths of targets in the first group of experiments.

	*EN*	*AG*	*STD*
Visible light	6.7775	27.4691	39.5135
Short-wave infrared	7.8693	25.7213	64.2759
Long-wave infrared	7.6096	52.5478	86.0797

**Table 3:** Evaluation indexes of intensity camera target images in the second group of experiments.

	*EN*	*AG*	*STD*
Visible light	6.3794	2.5526	24.8729
Short-wave infrared	6.8030	6.4061	34.4806
Long-wave infrared	6.9725	6.6710	32.7915

**Table 4:** Evaluation indexes of DOP images of different wavelengths of targets in the second group of experiments.

	*EN*	*AG*	*STD*
Visible light	6.8032	9.1349	39.3271
Short-wave infrared	7.8416	22.8076	61.0610
Long-wave infrared	7.9237	39.2035	70.0267

We take the evaluation metrics of the intensity camera imaging as the baseline to calculate the improvement rate of the image quality produced by using the Multi-Polarization Aerosol (MPA) imaging system with haze and multi-band polarization capabilities. Taking the example of the image standard deviation (STD) in the visible light wavelength at a visibility of 1km, the percentage improvement of the image quality produced by the MPA system compared to the intensity camera is: (39.5135–12.6645) / 12.6645 = 2.120 = 212.0%.The results indicate that the image evaluation metrics have improved across different spectral bands and detection distances. The maximum improvement rate for EN is 33.66%, while AG can be improved by over 200%, and the improvement rate for STD also reaches over 50%. It can be seen that the addition of the MPA-based system can significantly enhance image quality and highlight target details.

## Conclusion

Based on the multi-spectral polarization imaging technology of MPA, a haze permeable multi-spectral polarization imaging detection system is designed. By combining polarization imaging experiments in simulated indoor and real outdoor environments, it was found that an imaging system with the addition of micro-polarization array (MPA) has greater advantages in imaging targets in smoky environments and possesses strong smoke penetration capabilities (S1–S9 Figs). Through the analysis of EN, AG, and STD of polarized images in different spectral bands, it has been determined that the incorporation of MPA in the imaging system significantly enhances the polarization detection effectiveness for targets at different detection distances and across different spectral bands. The maximum improvement rate for EN is 33.66%, and AG showing an improvement of over 200%, and STD achieving an improvement rate of over 50% (S1–S4 Tables). This enhancement can highlight the target contours and enrich the target detail information. This article focuses on imaging static targets indoors and outdoors, and emphasizes that real-time detection of dynamic targets in multiple spectral bands will be the future development direction. The application of this technology can be utilized in areas such as detector integration and industrial manufacturing, providing new technical support for fields like military reconnaissance, fire and rescue operations, and urban surveillance.

## Supporting information

S1 Fig Target image without MPA in visible smoke environment. (PDF)

S2 Fig Target images obtained using a haze transmission polarization imaging detection system in a visible smoke environment. (PDF)

S3 Fig Comparison of contrast between target image with MPA and without MPA. (PDF)

S4 Fig Comparison of unbiased external field and polarization experimental images in the visible range under foggy weather conditions. (PDF)

S5 Fig Comparison of unbiased external field and polarization experimental images in the short-wave infrared range under foggy weather conditions. (PDF)

S6 Fig Comparison of unbiased external field and polarization experimental images in the long-wave infrared range with foggy weather conditions. (PDF)

S7 Fig Comparison of visible light unbiased external field and polarization experimental images in foggy weather condition. (PDF)

S8 Fig Comparison of short-wave infrared unbiased external field and polarization experimental images under foggy weather conditions. (PDF)

S9 Fig Comparison of long-wave infrared unbiased external field and polarization experimental images under foggy weather conditions. (PDF)

S1 Table Evaluation indexes of intensity camera target images in the first group of experiments. (PDF)

S2 Table Evaluation indexes of DOP images of different wavelengths of targets in the first group of experiments. (PDF)

S3 Table Evaluation indexes of intensity camera target images in the second group of experiments. (PDF)

S4 Table Evaluation indexes of DOP images of different wavelengths of targets in the second group of experiments. (PDF)
