# Supplementary figures and images for: Analysis of multispectral polarization imaging image information based on micro-polarizer array
Source: PLoS One. 2024 Jan 30;19(1):e0296397. doi: 10.1371/journal.pone.0296397 (PMC10826961; doi:10.1371/journal.pone.0296397)

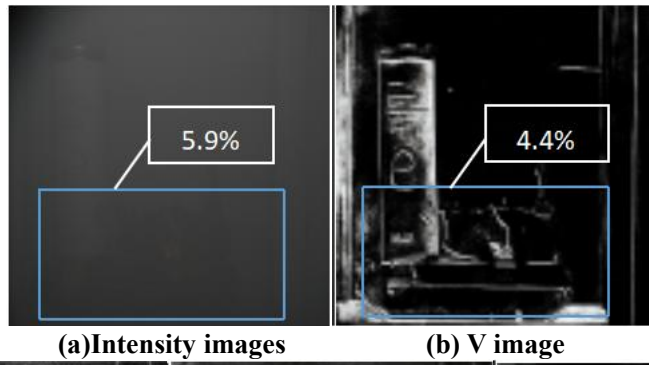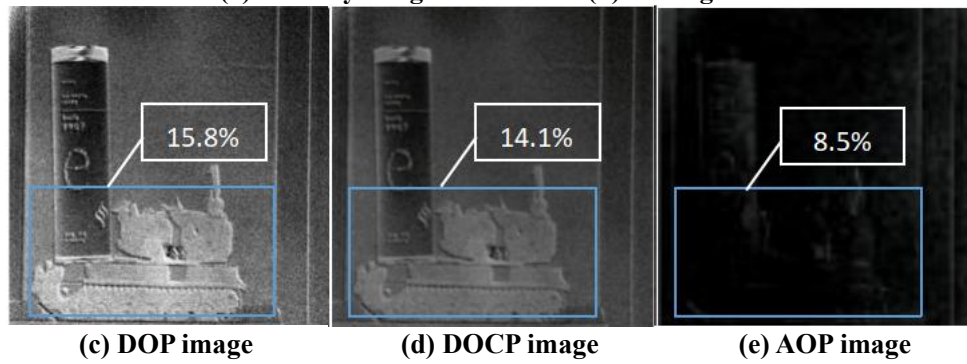

S1 Fig. Target image without MPA in visible smoke environment

Supplement: S1 Fig — (PDF) [file pone.0296397.s001.pdf]

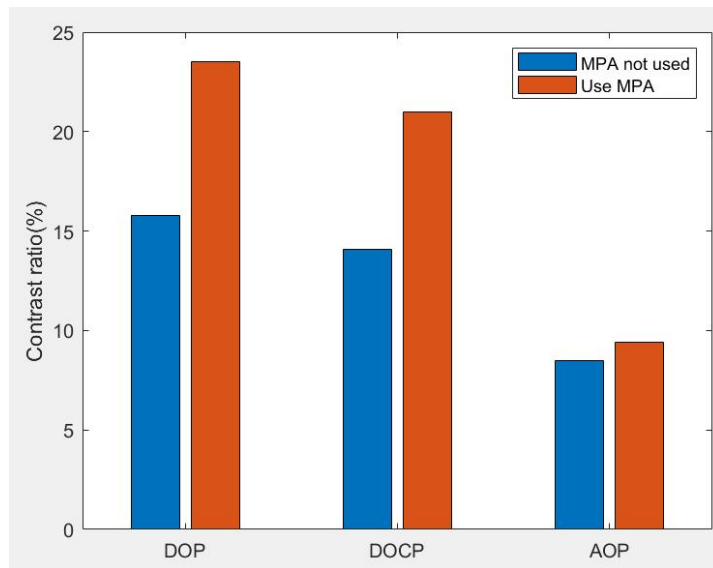

**S3 Fig. Comparison of contrast between target image with MPA and without MPA.**

Supplement: S3 Fig — (PDF) [file pone.0296397.s003.pdf]
