# Supplementary material for: Analysis of multispectral polarization imaging image information based on micro-polarizer array
Source: PLoS One. 2024 Jan 30;19(1):e0296397. doi: 10.1371/journal.pone.0296397 (PMC10826961; doi:10.1371/journal.pone.0296397)
Supplement: S2 Fig — (PDF) [file pone.0296397.s002.pdf]

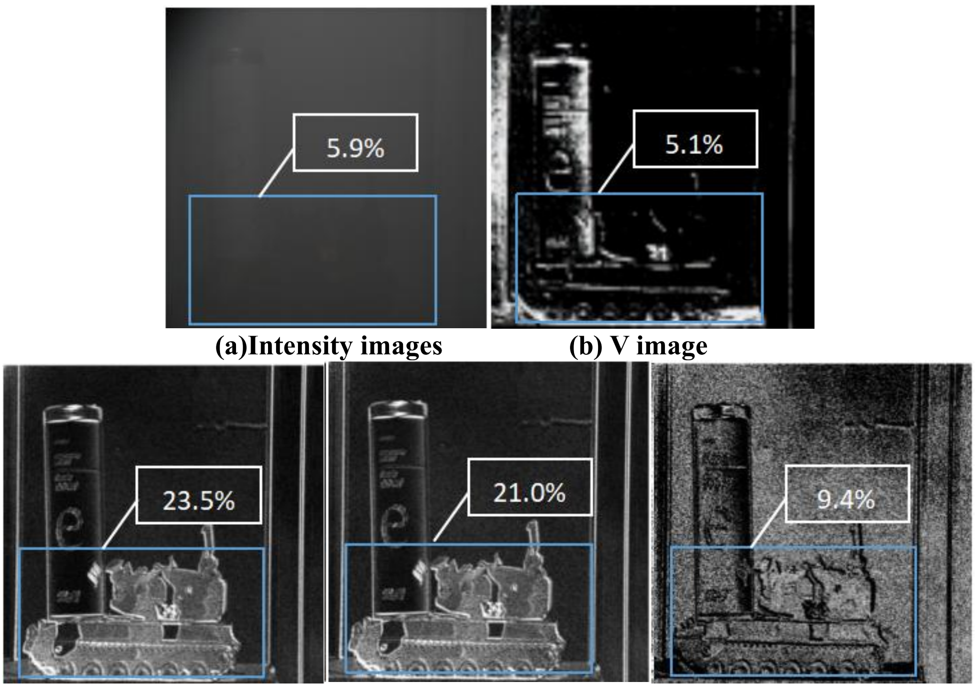

(a)Intensity images (b) V image

(c) DOP image (d) DOCP image (e) AOP image

S2 Fig. Target images obtained using a haze transmission polarization imaging detection system in a visible smoke environment
