# Supplementary material for: Analysis of multispectral polarization imaging image information based on micro-polarizer array
Source: PLoS One. 2024 Jan 30;19(1):e0296397. doi: 10.1371/journal.pone.0296397 (PMC10826961; doi:10.1371/journal.pone.0296397)
Supplement: S4 Fig — (PDF) [file pone.0296397.s004.pdf]

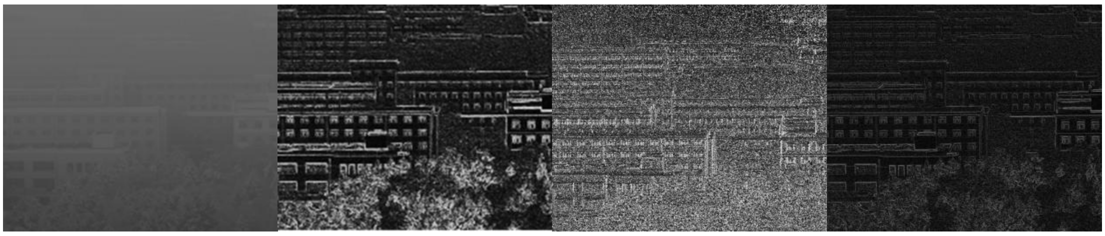

(a) Normal camera intensity (b) DOP (c) AOP (d) DOCP

S4 Fig. Comparison of unbiased external field and polarization experimental images in the visible range under foggy weather conditions.
