# Supplementary material for: Analysis of multispectral polarization imaging image information based on micro-polarizer array
Source: PLoS One. 2024 Jan 30;19(1):e0296397. doi: 10.1371/journal.pone.0296397 (PMC10826961; doi:10.1371/journal.pone.0296397)
Supplement: S7 Fig — (PDF) [file pone.0296397.s007.pdf]

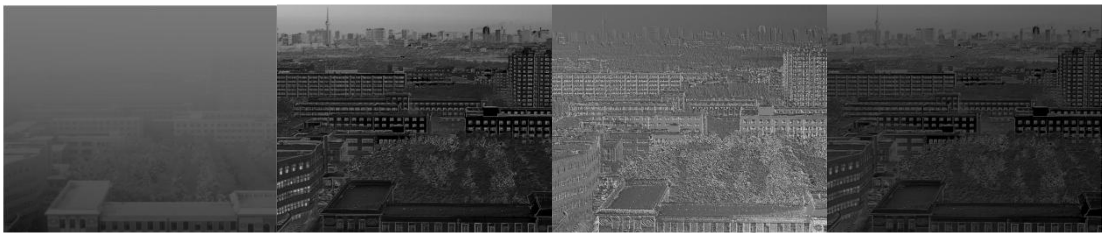

(a) Normal camera intensity (b) DOP (c) AOP (d) DOCP

S7 Fig. Comparison of visible light unbiased external field and polarization experimental images in foggy weather condition.
