# Supplementary material for: Analysis of multispectral polarization imaging image information based on micro-polarizer array
Source: PLoS One. 2024 Jan 30;19(1):e0296397. doi: 10.1371/journal.pone.0296397 (PMC10826961; doi:10.1371/journal.pone.0296397)
Supplement: S1 Table — (PDF) [file pone.0296397.s010.pdf]

S1 Table. Evaluation indexes of intensity camera target images in the first group of experiments

	EN	*AG*	*STD*
Visible light	5.4086	1.3845	12.6645
Short-wave infrared	6.0228	5.6561	18.3213
Long-wave infrared	7.6094	15.2762	50.5361
