# Supplementary material for: Analysis of multispectral polarization imaging image information based on micro-polarizer array
Source: PLoS One. 2024 Jan 30;19(1):e0296397. doi: 10.1371/journal.pone.0296397 (PMC10826961; doi:10.1371/journal.pone.0296397)
Supplement: S2 Table — (PDF) [file pone.0296397.s011.pdf]

S2 Table. Evaluation indexes of DOP images of different wavelengths of targets in the first group of experiments

	EN	*AG*	*STD*
Visible light	6.7775	27.4691	39.5135
Short-wave infrared	7.8693	25.7213	64.2759
Long-wave infrared	7.6096	52.5478	86.0797
