# Supplementary material for: Analysis of multispectral polarization imaging image information based on micro-polarizer array
Source: PLoS One. 2024 Jan 30;19(1):e0296397. doi: 10.1371/journal.pone.0296397 (PMC10826961; doi:10.1371/journal.pone.0296397)
Supplement: S3 Table — (PDF) [file pone.0296397.s012.pdf]

S3 Table. Evaluation indexes of intensity camera target images in the second group of experiments

	EN	*AG*	*STD*
Visible light	6.3794	2.5526	24.8729
Short-wave infrared	6.8030	6.4061	34.4806
Long-wave infrared	6.9725	6.6710	32.7915
