# Supplementary material for: Analysis of multispectral polarization imaging image information based on micro-polarizer array
Source: PLoS One. 2024 Jan 30;19(1):e0296397. doi: 10.1371/journal.pone.0296397 (PMC10826961; doi:10.1371/journal.pone.0296397)
Supplement: S4 Table — (PDF) [file pone.0296397.s013.pdf]

S4 Table. Evaluation indexes of DOP images of different wavelengths of targets in the second group of experiments

	EN	*AG*	*STD*
Visible light	6.8032	9.1349	39.3271
Short-wave infrared	7.8416	22.8076	61.0610
Long-wave infrared	7.9237	39.2035	70.0267
